# Hazards and Health Risks of the Antibacterial Agent Triclosan to Fish: A Review

**DOI:** 10.3390/jox15060204

**Published:** 2025-12-02

**Authors:** Jiangang Wang, Nannan Ma, Gancong Mo, Xian Qin, Jin Zhang, Xiangping Yao, Jiahua Guo, Zewei Sun

**Affiliations:** 1 Guangdong Provincial Key Laboratory of Marine Disaster Prediction and Prevention, Shantou University, Shantou 515063, China; 23jgwang@stu.edu.cn (J.W.); 21nnma@alumni.stu.edu.cn (N.M.); 24jzhang@stu.edu.cn (J.Z.); 2 College of Engineering, Guangzhou College of Technology and Business, Guangzhou 510850, China; mogancong@163.com (G.M.); xp_yao921@163.com (X.Y.); 3 State Key Laboratory of Marine Environmental Health, City University of Hong Kong, Kowloon, Hong Kong SAR, China; xianqin2@cityu.edu.hk; 4 Shaanxi Key Laboratory of Earth Surface System and Environmental Carrying Capacity, College of Urban and Environmental Sciences, Northwest University, Xi’an 710127, China; jiahua_guo@nwu.edu.cn

**Keywords:** pharmaceuticals and personal care products, triclosan, developmental toxicity, long-term effects, fish, ecological risk assessment

## Abstract

Triclosan (TCS) is a widely used antimicrobial agent found in personal care products and household cleaners. While valued since the 1960s for its ability to inhibit bacterial fatty acid synthesis, its environmental persistence, ecotoxicity, and bioaccumulative potential have raised significant global concern. The increased use of disinfectants during the COVID-19 pandemic has further exacerbated its prevalence as an aquatic pollutant. In the environment, TCS is distributed through water bodies and sediments, undergoing processes such as biodegradation and photochemical degradation. Its bioaccumulation poses a substantial threat to aquatic organisms, particularly fish. A growing body of research indicates that TCS acts as an endocrine disruptor and developmental toxicant, with documented adverse effects encompassing impaired embryonic and larval development, skeletal malformations, and induction of oxidative stress, mitochondrial dysfunction, DNA damage, and inflammatory responses. Furthermore, TCS exposure is linked to reproductive toxicity, including altered sex hormone levels and diminished reproductive capacity. This review consolidates current knowledge on the chemical properties, environmental fate, biodegradation pathways, and ecotoxicological impacts of TCS, with a specific emphasis on its multifaceted health risks to fish. The synthesis aims to provide a foundation for future research, inform environmental risk assessments, and support the development of evidence-based regulatory measures.

## 1. Introduction

Triclosan (TCS) is a chlorinated aromatic compound with broad-spectrum antibacterial activity. Its mechanism of action involves inhibiting bacterial fatty acid synthesis and disrupting cell membranes [1,2,3,4], which led to its initial adoption as a hospital bactericide. Historically, TCS was incorporated as a preservative and antimicrobial agent into a vast array of over 2000 consumer products, including soaps, toothpastes, cosmetics, textiles, kitchenware, and children’s toys. This pervasive use resulted in TCS becoming a ubiquitous environmental contaminant, primarily entering waterways through wastewater treatment plant effluents and sewage sludge application on agricultural land [5].

Regulatory responses to health and environmental concerns have varied globally. The U.S. Food and Drug Administration and the European Union banned the use of TCS in over-the-counter antibacterial soaps in 2016. However, this ban is not worldwide, and TCS remains a key ingredient in many other personal care products, such as toothpaste and hand sanitizers, at concentrations typically between 0.1% and 1% (*w*/*w*) [2,3,6,7]. This variance in global regulation has led to known environmental hotspots, with elevated TCS concentrations detected in waters receiving effluent from manufacturing plants, urban centers, and hospitals [8,9]. This extensive use and environmental resilience have led to the widespread detection of TCS in rivers, lakes, and coastal waters globally. Notably, environmental concentrations surged during the COVID-19 pandemic due to heightened disinfectant consumption [10], underscoring the direct link between consumer use and environmental loading and highlighting its continued relevance as a pollutant of concern.

Structurally, TCS contains two benzene rings with a high chlorine content, conferring properties similar to persistent pollutants like polychlorinated biphenyls and dioxins [11]. This structural analogy suggests that TCS may also share the tendencies of these classic pollutants for bioaccumulation and biomagnification, raising concerns for long-term ecosystem exposure. Over 90% of used TCS enters wastewater systems, and conventional treatment processes remove it with limited efficiency; approximately 30% is ultimately discharged into water bodies on a global scale. It is routinely detected in wastewater influent and effluent, sludge, surface water, and sediments (Table 1). Characterized by low volatility (vapor pressure: 4 × 10^−6^ mm Hg at 20 °C), high lipophilicity (logKow: 4.8), and a long half-life up to several months, TCS persists in surface water at ng/L concentrations, where it remains stable and bioaccumulates [12,13,14]. In severely contaminated areas, concentrations can reach 40 μg/L, underscoring significant environmental concern [15].

In aquatic systems, TCS primarily undergoes photodegradation and biodegradation. Photochemical reactions can transform TCS into hazardous derivatives, including 2,8-dichlorodibenzo-p-dioxin, 2,7-dichlorodibenzo-p-dioxin, and hydroxylated dichlorodibenzofurans, which may pose greater risks than the parent compound [39,40]. Notably, these dioxin and furan derivatives are of particular concern as they are generally more persistent, bioaccumulative, and significantly more toxic than the parent TCS, with well-documented potential for carcinogenic, endocrine-disrupting, and developmental effects [41]. Biodegradation represents another critical pathway. Fungal laccase/redox mediator systems can enzymatically transform TCS by cleaving its diphenyl ether bond, reducing its toxicity [42]. Certain bacterial species, such as *Pseudomonas putida* and *Alcaligenes xylosoxidans*, can utilize TCS as a carbon source for degradation with an efficiency up to 98% [43,44].

**Figure 1:**
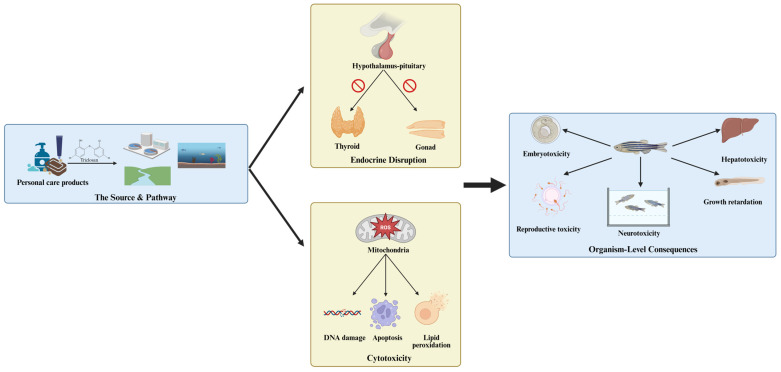
A schematic diagram showing the sources, environmental occurrence, and ecotoxicity of triclosan.

Due to its prevalence and persistence, TCS is ranked among the top ten pollutants in terrestrial and aquatic environments [45,46] and bioaccumulates in organisms across trophic levels, from algae to marine mammals [16]. The bioaccumulation of TCS in marine mammals indeed highlights a potential public health concern, as humans, particularly high-level seafood consumers, may be inadvertently exposed to this compound through the diet [47]. While the direct acute toxicity from dietary exposure is likely low, chronic intake has been linked to potential endocrine-disrupting effects and contributions to antimicrobial resistance in experimental models [48,49]. Crucially, maximum residue limits for TCS in food are not consistently established globally. Ecotoxicological studies (Table 2 and Table 3) demonstrate that acute and sub-chronic TCS exposure induces a range of adverse effects in aquatic organisms. These include the induction of oxidative stress, disruption of lipid metabolism, DNA damage, and various histopathological changes. These effects can result in embryonic developmental abnormalities, endocrine disruption, neurotoxicity, behavioral alterations, and reproductive impairment (Figure 1) [50,51]. Notably, although many studies have revealed that triclosan exposure in fish causes thyroid dysfunction, metabolic disorders, reduced fertility, and liver damage, these adverse effects are mostly observed at high concentrations [50,52,53]. In contrast, multiple investigations, including Mihaich [54], demonstrate that at the lower (<90 μg/L), environmentally relevant concentrations of triclosan found in water and sediments (Table 1), no significant impacts on the HTP-axis, liver, metabolism, or reproductive outcomes are observed. Furthermore, TCS degradation products formed under oxidative conditions may also pose risks to aquatic life, though their toxicity to fish remains inadequately studied. Therefore, future research must prioritize the identification, monitoring, and toxicological assessment of these transformation products to fully understand the ecological risks posed by TCS throughout its lifecycle.

## 2. Methods

**Figure 2:**
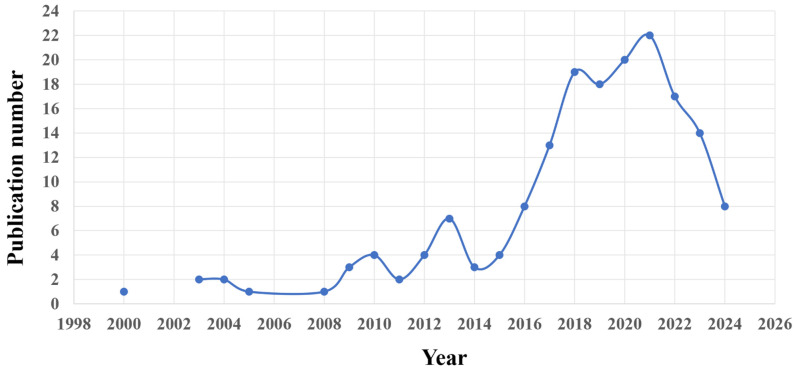
Publications related to triclosan-induced toxicity in fish in the literature. Literature searching was performed on Web of Science, and relevant papers published during 1998–2024 were sourced. The first related study recorded on Wed of Science was published in 2000.

To comprehensively evaluate the latest advances in understanding the effects of triclosan on fish, literature searches were conducted by three coauthors. The search encompassed publications from January 1998 to December 2024. Primary databases, including PubMed, Web of Science (Figure 2), and Google Scholar, were queried using the key term “triclosan fish” or “triclosan toxicity fish”. The resulting articles in English were selected for in-depth reading based on their relevance to the titles and abstracts. The search strategy was supplemented by a manual examination of reference lists from these relevant articles to identify additional studies. In total, 103 references were selected for final review.

## 3. Metabolism and Cytotoxicity of Triclosan

TCS can enter fish through dermal contact, diet, and aqueous exposure, undergoing a typical two-phase metabolic process: Phase I Metabolism (oxidation, reduction, hydrolysis) and Phase II Metabolism (conjugation reactions) [5,9,85]. Phase I Metabolism can be further divided into hydroxylation, ether bond cleavage, cyclization, and dechlorination. Hydroxylation involves the introduction of a hydroxyl (-OH) group onto the benzene ring of TCS, potentially occurring on the less chlorinated ring to form monohydroxy-triclosan (TP 302). Ether bond cleavage, one of the primary metabolic pathways, involves the breakage of the ether bond (C-O-C) at the C-6 position, generating metabolites such as 2,4-dichlorophenol (TP 160). TCS may also undergo cyclization to form dioxin-like derivatives, which are of significant concern due to their high ecotoxicological potential. These include compounds such as hydroxylated dichlorodibenzodioxin (DCDD, P4). Dechlorination at the C-3 position produces di-chloro-hydroxydiphenyl ether (TP 252). These processes significantly increase the polarity of TCS, preparing it for subsequent metabolism. Phase II Metabolism typically involves the addition of polar groups (e.g., sulfate, glucuronic acid, or glutathione) to Phase I metabolites, further enhancing their water solubility and promoting excretion with an efficiency larger than 80% [82]. Phase II reactions include sulfation and glucuronidation. TCS or its hydroxylated metabolites may undergo sulfation to form sulfated metabolites, such as 2,4-dichlorophenol-O-sulfate (TP 240), triclosan-O-sulfate (TP 366), and monohydroxy-triclosan-O-sulfate (TP 382). Glucuronidation involves the addition of a glucuronic acid group to hydroxylated metabolites, increasing their water solubility and facilitating excretion [7,86].

While these metabolic pathways are primarily detoxification mechanisms, they can also produce reactive intermediates whose presence directly connects to the observed cytotoxic effects. Mitochondria generate ATP via the electron transport chain, but TCS can interfere with the electron transport chain, increasing electron leakage and contributing to the generation of reactive oxygen species (ROS) such as superoxide. Simultaneously, it reduces the activity of key mitochondrial antioxidant enzymes (e.g., SOD, GPx), weakening the cell’s ability to clean ROS. The resulting ROS accumulation triggers oxidative stress, further leading to mitochondrial dysfunction, decreased membrane potential, impaired ATP synthesis, and induction of apoptosis via activation of the MAPK/p53 and Bax/Bcl-2/caspase pathways. It also promotes DNA damage and inflammatory responses, constituting the primary cytotoxic mechanisms of TCS. ROS can activate multiple apoptotic signaling pathways, including intrinsic and extrinsic pathways. ROS promote the expression of Bax and Bak, increase mitochondrial membrane permeability, release cytochrome C, and activate the caspase cascade, leading to apoptosis. Experiments show that TCS exposure significantly increases the number of TUNEL-positive cells in the hearts of zebrafish larvae (250 μg/L from 30 to 120 days post fertilization under the conditions of a 14 h: 10 h light: dark cycle and temperature of 28 ± 0.5 °C) and in the olfactory epithelium and olfactory bulbs of goldfish, while decreasing Ca^2+^-ATPase and Na^+^/K^+^-ATPase activity and increasing caspase-1 and caspase-3 activity [6,87]. Zebrafish embryos exposed to TCS (50 and 250 μg/L) from 4 h post-fertilization to 96 h also showed significant apoptosis [88]. In reproductive organs, high concentrations of TCS (85, 170 µg/L) led to TUNEL-positive signals in ovarian oocytes and testicular Sertoli cells and spermatocytes, along with downregulation of antioxidant genes (*sod*, *gpx1a*, and *cat*), upregulation of apoptosis-related genes (*bax* and *p53*), and a decrease in Bcl-2 [89,90]. These findings indicate that TCS can induce oxidative stress and apoptosis in key reproductive cells, which is highly likely to impair individual fertility and, at a population level, could lead to reduced reproductive success in wild fish populations. In vitro exposure of zebrafish liver cells to TCS (0.5–2 mg/L) for 24 h showed apoptotic cell proportions of 21%, 36%, and 80%, respectively [91]. Mechanistically, TCS increases ROS to activate oxidative stress, further inducing MAPK (ERK, JNK, and p38) and p53 signaling pathways. The imbalance in Bax/Bcl-2 leads to cytochrome C release, activating effector caspases such as caspase-3, ultimately triggering apoptosis [11,91].

**Figure 3:**
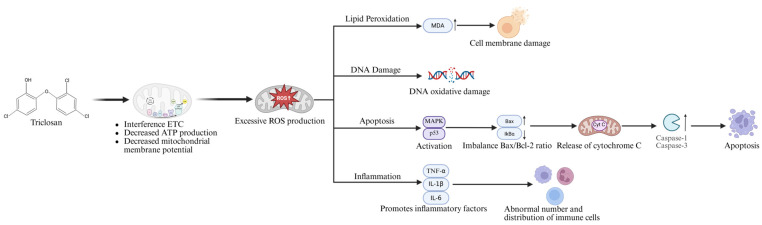
Molecular mechanism diagram of triclosan-induced cytotoxicity in fish. MDA: malondialdehyde, MAPK: mitogen-activated protein kinase, p53: tumor protein 53, TNF-α: tumor necrosis factor-alpha, IL-1β: interleukin-1 beta, IL-6: interleukin-6, ETC: electron transfer chain, ROS: reactive oxygen species. An upward and downward arrow indicates increase and decrease, respectively.

ROS can react with intracellular lipids, proteins, and DNA, causing oxidative damage. Lipid peroxidation (LPO) damages membrane fluidity and integrity. Malondialdehyde (MDA), a primary marker of LPO, reflects oxidative stress levels. Sustained LPO damages cell membranes by impairing their fluidity and integrity, which disrupts critical functions like ion transport, signal transduction, and organelle compartmentalization, ultimately leading to a loss of cellular homeostasis. Experiments show that TCS exposure significantly increases MDA and LPO levels in fish. In zebrafish intestinal tissue, 250 μg/L TCS elevated MDA [92]. In zebrafish larvae, MDA content increased positively with TCS concentration [93]. In zebrafish liver, 189 µg/L TCS increased LPO by approximately 40%, while the same concentration exposure in South American catfish gills resulted in a 13% increase in LPO [71]. In *Anabas testudineus* hepatocytes and goldfish liver homogenate, low concentrations of TCS (9 µg/L and 0.28–0.56 mg/L) also significantly elevated MDA levels [26]. Notably, the induction of oxidative stress in *Anabas testudineus* at a much lower concentration (9 µg/L) compared to the goldfish model (280–560 µg/L) suggests a potentially higher sensitivity in this species, which could be attributed to differences in metabolic capacity or antioxidant defense systems. These results indicate that TCS induces oxidative stress and cellular damage (Figure 3).

Mitochondria are both a primary site of ROS generation and a key target of ROS attack. TCS exposure can lead to mitochondrial dysfunction in fish (*Anabas testudineus*) and cell models (zebrafish liver cells ZF-L), manifested as decreased ATP production, enhanced oxidative stress, reduced antioxidant enzyme activity, decreased levels of reduced glutathione, and lowered mitochondrial membrane potential (MMP)—a particularly sensitive early indicator of toxicity that often precedes irreversible commitment to cell death [91]. In zebrafish, detection of MMP in larvae showed weakened red fluorescence in the 50 and 250 μg/L TCS treatment groups, indicating decreased MMP [53]. Excessive ROS formation also causes oxidative DNA damage, including base oxidation, single-strand and double-strand breaks, further activating apoptotic pathways. Micronucleus tests and comet assays show that TCS significantly increases micronucleus formation and DNA damage in fish erythrocytes and hepatocytes. For example, DNA damage significantly increased in yellow eels exposed to TCS under low pH conditions [78]. Micronucleus tests observed a significant increase in the number of micronuclei and binucleated cells in erythrocytes of African sharptooth catfish (*Clarias gariepinus*) exposed to TCS [52]. In zebrafish, the extent of DNA damage was quantified by measuring the fluorescence intensity of comet tails. Following TCS treatment at 0.5596 mg/L, tail moment in hepatocytes profoundly increased compared to the solvent control group, indicating TCS-induced DNA damage. Tail length, tail moment, and tail DNA percentage in zebrafish hepatocyte comet assays increased with TCS concentration [56,69]. Comet assay results on zebrafish embryos showed that exposure to 1 μg/L of TCS led to a significant increase in the frequency of DNA damage at the end of 120 h of exposure, indicating slight primary genotoxic effects [94]. Furthermore, Indian major carp (*Labeo rohita*) exposed to TCS showed a significant increase in DNA damage, which remained higher than the control group even during the 10-day recovery period, suggesting the potential persistence of TCS genotoxicity [25,95]. These results indicate that TCS, by inducing mitochondrial dysfunction and ROS accumulation, not only damages cellular energy metabolism but also triggers oxidative stress and DNA damage, severely impacting cell survival and genetic stability.

Excessive ROS formation can promote the expression of inflammatory factors such as TNF-α, IL-1β, and IL-6. These factors interact with ROS, forming pathological positive feedback that exacerbates cellular damage and apoptosis. Studies have found that TCS exposure interferes with the development and function of immune cells in zebrafish, altering the balance between pro-inflammatory and anti-inflammatory factor expression [50,68]. Under 250 μg/L TCS treatment, the number and distribution of neutrophils, macrophages, and T cells in zebrafish quantified by bioimaging under a fluorescent microscope were abnormal, and the expression of immune-related genes and proteins significantly changed, including upregulation of pro-inflammatory factors and downregulation of anti-inflammatory factors [67]. Studies on striped catfish showed that TCS exposure led to decreased respiratory burst activity, myeloperoxidase activity, and phagocytic activity, indicating impaired immune defense function [80]. Additionally, mucus deposition on the body surface of the freshwater fish *Anabas testudineus* increased and rose with TCS concentration (0.009, 9, and 176.7 μg/L), reflecting the fish immune system’s response to exogenous toxic substances by enhancing mucus secretion [14]. While mucus serves as a critical first-line immune barrier, its hyper-secretion in this context is more indicative of a chronic stress response. The sustained, dose-dependent increase signals a persistent toxic challenge that forces the fish to expend significant energy on defense, potentially leading to immunosuppression and reduced fitness over time. In summary, TCS exposure can perturb the fish immune system through ROS-mediated pro-inflammatory responses, manifesting as both decreased immune cell function and increased defensive mucus secretion, forming complex immunomodulatory effects.

ROS accumulation can affect cell survival and death by activating the MAPK signaling pathway (including JNK, p38, and ERK). Studies show that high-concentration TCS exposure (125–250 μg/L) causes chondrocyte swelling, deformation, reduced numbers, and nuclear displacement, along with a significant decrease in bone-derived alkaline phosphatase activity, suggesting inhibited osteoblast activity and weakened bone mineralization, thereby affecting larval bone formation. At the molecular level, TCS exposure downregulated skeletal development-related genes that play key roles in cartilage formation (e.g., *sox4a*, *sox11b*, *sox9a*, *col2a1a*,), bone mineralization (e.g., *spp1*, *bglap*, *bmp2a*, *bmp4*), and osteoblast differentiation (e.g., *hoxb3a*, *runx2b*) [13]. Furthermore, TCS exposure caused abnormal miRNA expression, specifically upregulation of miR-144, miR-34a, and miR-153a-5p, and downregulation of miR-34b, miR-200b-5p, and miR-21. These miRNA changes may participate in the regulation of oxidative stress responses and skeletal development by modulating their target genes through mRNA degradation or translation suppression, providing molecular mechanistic support for TCS-induced bone development abnormalities [26,93]. In summary, TCS interferes with fish skeletal development and mineralization processes through synergistic multi-pathway actions, including induction of oxidative stress, inhibition of osteogenesis-related genes, and alteration of miRNA expression.

## 4. Embryonic Development

### 4.1. Embryotoxicity

Fish embryos are highly sensitive to environmental pollutants, in addition to transparency, rapid development, ecological relevance, making it a common model for assessing the toxicity of chemical substances. Studies have shown that TCS exhibits endocrine-disrupting effects and significant toxicity to fish embryos [69]. Qian et al. (2024) found that 250 μg/L TCS significantly reduced the hatching rate of zebrafish embryos [68]. Ning Tang et al. further reported that zebrafish embryos treated with 30–900 ng/mL TCS showed a decline in hatching rate from 34.5% to 4.3%, with all larvae dying within 24 h at 900 μg/L [66]. Annamaria Iannetta observed a dose-dependent delay in zebrafish embryo development and hatching after 72 h post-fertilization (hpf) [84]. Under high-concentration exposure (1.60–4.16 mg/L), the hatching rate decreased to approximately 68–70%, while the proportion of developmental abnormalities reached 43–46% [52]. Similarly, Nile tilapia exposed to 2.092 mg/L TCS exhibited 100% mortality within 24 h, while no mortality was observed at lower concentrations (0.131–0.262 mg/L) [70]. This rapid and complete mortality at a relatively high concentration demonstrates a clear acute toxicological threshold and underscores the potential for TCS to cause severe, immediate population crashes in the event of a spill or point-source pollution event in aquatic environments. The mortality rate of zebrafish embryos increased significantly with time and dose, with significant increase after 96 h of exposure to 0.4 mg/L and higher concentrations of TCS [61].

In addition to changes in hatching and survival rates, TCS also caused noticeable embryonic morphological abnormalities, including melanin deficiency, yolk sac enlargement, spinal deformities, and cardiac hemorrhage. The malformation rate under acute exposure reached 27.4% [96,97]. Zebrafish embryos exposed to 0.9 mg/L TCS showed significant delays in otolith formation and ocular pigmentation [12]. The disruption of these processes is particularly significant; otoliths are vital for the vestibular system (governing balance and orientation), while timely ocular pigmentation is crucial for the development of functional vision. Furthermore, surviving larvae exposed to 0.5 mg/L TCS exhibited spinal deformities and pericardial edema [12]. The edema rate, an important indicator of TCS embryotoxicity, increased significantly as the exposure pH decreased from 8 to 6 (pH 6: 17.5%, pH 7: 14.7%, and pH 8: 14.1%) [98]. Furthermore, the severity of yolk sac edema was enhanced in TCS-exposed larvae (from 6 to 72 h post fertilization), and their average body length was significantly shorter compared to the control group [68].

### 4.2. Developmental Toxicity

Following the impact of TCS on embryonic development, fish growth and development also exhibit significant toxic effects. Studies have shown that TCS exposure increases mucus secretion in the skin and gills of fish such as Mozambique tilapia (*Oreochromis mossambicus*), striped catfish (*Pangasianodon hypophthalmus*), and guppy (*Poecilia reticulata*) [70,79,99]. Mucus secretion is a natural defense response of fish to environmental pollutants, pathogens, and physical damage. TCS exposure stimulates mucus secretion to form a physical barrier, reducing the penetration and absorption of harmful substances.

Furthermore, growth indicators of fish vary significantly under different experimental conditions. Qian et al. (2024) observed that zebrafish exposed to 125 and 250 μg/L TCS between 30 and 100 dpf showed significant reductions in body weight, length, and width [68]. In contrast, Danting Wang found that exposure to a mixture of TCS and its derivatives (TCS-DT) significantly increased the body mass index and width of zebrafish at 90 days [100]. In a 3-month chronic TCS exposure experiment on adult zebrafish, significant increases in body weight and length were observed [15]. These results indicate that the effects of TCS on fish development are complex and depend on dose, duration, and species.

**Figure 4:**
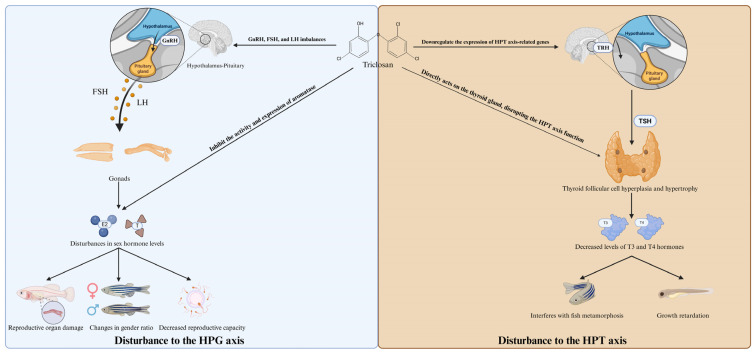
A schematic diagram illustrating triclosan-induced endocrine disruption in fish. GnRH: gonadotropin-releasing hormone, FSH: follicle-stimulating hormone, LH: luteinizing hormone, TSH: thyroid-stimulating hormone, HPG axis: hypothalamic–pituitary–gonadal axis, HPT axis: hypothalamic–pituitary–thyroid axis, T3: triiodothyronine, T4: thyroxine, E2: 17β-estradiol, T: testosterone.

The molecular structure of TCS resembles several nonsteroidal estrogens, such as diethylstilbestrol and bisphenol A, due to its two phenolic hydroxyl functional groups. This suggests that TCS has the potential to act as an endocrine disruptor [101]. Specifically, it can interfere with the hypothalamic–pituitary–gonadal (HPG) axis, leading to altered sex hormone levels, impaired gonadal development, reduced fertility, and the induction of vitellogenin in male fish—a clear marker of estrogenic exposure. These disruptions can ultimately result in skewed sex ratios, abnormal reproductive behavior, and diminished reproductive success at the population level. Indeed, some studies have shown that TCS exposure activates the HPG axis, increasing the levels of hormones such as GnRH, FSH, and LH, thereby affecting the production and regulation of sex hormones (Figure 4). This may ultimately impact fish reproductive health, including reproductive capacity and the sexual development of offspring [30,58]. TCS exposure upregulated GnRH mRNA expression in the hypothalamus, indicating that TCS may stimulate the hypothalamus to produce more GnRH [56,58]. Consistent with upregulation of GnRH mRNA expression, GnRH levels in the serum of Yellow River carp (*Cyprinus carpio*) and zebrafish also increased after TCS treatment [56]. However, in a 42-day exposure experiment on 7-week-old Yellow River carp using different concentrations of TCS (0, 0.04, 0.08, and 0.16 mg/L), a decrease in GnRH mRNA expression in the hypothalamus, particularly in the 0.08 and 0.16 mg/L TCS treatment groups was found, where cGnRH-II-2 mRNA levels and GnRH concentrations were reduced [30]. The observed contradiction, where low concentrations of TCS increase GnRH while higher concentrations decrease it, is a hallmark of endocrine disruption. A plausible mechanism involves a compensatory negative feedback loop. Initially, low-level disruption may stimulate a compensatory surge in GnRH release. However, at higher concentrations, the sustained disruption could trigger a powerful negative feedback response from downstream hormones (e.g., elevated cortisol or sex steroids), ultimately suppressing hypothalamic GnRH synthesis and release to prevent systemic overload.

Simultaneously, TCS exposure increased the mRNA expression of the β-subunits of follicle-stimulating hormone (FSH) and luteinizing hormone (LH) in the pituitary, indicating that TCS stimulates the anterior pituitary to produce more FSH and LH [56,58]. This heightened gonadotropin signaling directly promotes gonadal maturation by stimulating steroidogenesis (the production of sex steroids like estrogen and testosterone) and driving the progression of gametogenesis, including vitellogenesis in females and spermatogenesis in males. In *Catla catla* treated with 0.073 mg/L TCS, FSH and LH concentrations increased significantly by 35.11% and 40.69%, respectively, compared to the control group [58]. However, Chokki Veettil et al. (2024) observed a concentration-dependent significant decrease in FSH and LH. The observed contradiction, where lower TCS doses increase FSH/LH while higher doses decrease them, is a hallmark of a disrupted hypothalamic-pituitary-gonadal (HPG) axis feedback loop [14]. A plausible mechanism involves an initial compensatory stimulation of gonadotropin release at low doses. However, at higher concentrations, this may trigger a powerful negative feedback response from downstream hormones (e.g., elevated sex steroids or cortisol), or lead to the downregulation of pituitary GnRH receptors, ultimately suppressing FSH and LH synthesis to prevent systemic hormonal overload. Gonadal hormone levels and ratios were also affected. The E2/T ratio—the balance between the primary female hormone, estradiol (E2), and the primary male hormone, testosterone (T)—is a critical indicator of sexual phenotype. Increases or decreases in this ratio can lead to hormonal imbalance, potentially causing intersex conditions, impaired gonadal development, and reduced fertility [30,56]. Chokki Veettil et al. (2024) showed that serum estradiol levels significantly decreased in female fish and increased in male fish, while testosterone levels significantly decreased in both sexes [14]. These opposite changes suggest a feminizing effect on males and a de-feminizing or suppressive effect on females. In males, elevated E2 can lead to the inappropriate production of vitellogenin (an egg yolk protein) and disrupt courtship behaviors, while the overall suppression of testosterone in both sexes can impair libido, gametogenesis, and secondary sexual characteristics, collectively threatening reproductive success. The primary reason is that TCS affects the activity of aromatase and the mRNA expression of cyp19a1a and cyp19a1b in the gonads and hypothalamus. These genes encode for the aromatase enzyme, but with distinct tissue-specific functions: cyp19a1a is predominantly expressed in the ovaries and is essential for converting androgens to estrogens to support oocyte growth and vitellogenesis, while cyp19a1b is primarily neuronal, expressed in the hypothalamus, and crucial for regulating neuroendocrine pathways that control sexual behavior and the feedback of the reproductive axis. Changes in their activity may lead to imbalances in sex hormones [14,30,56,58].

Additionally, TCS exposure inhibits the activity of 3β-hydroxysteroid dehydrogenase (3β-HSD) and 17β-hydroxysteroid dehydrogenase (17β-HSD), enzymes critical for sex hormone biosynthesis. The inhibition of 3β-HSD disrupts the conversion of pregnenolone to progesterone, a key early step in the steroidogenic pathway, while the suppression of 17β-HSD directly impairs the final activation step of androgens (like androstenedione to testosterone). Since testosterone is both a crucial end-product and the primary precursor for estradiol synthesis via aromatase, this dual inhibition provides a direct mechanistic explanation for the observed decreases in both testosterone and estradiol [14]. TCS exposure also significantly increased Er protein levels and mRNA expression in the liver of female carp, suggesting that TCS may enhance Er levels by increasing the transcriptional activity of the Er gene [56]. This upregulation is likely a pathological response to the endocrine-disrupting activity of TCS. By increasing Er expression, TCS could sensitize the liver to even low levels of circulating estrogen (or estrogen-mimics), leading to an exaggerated and dysregulated vitellogenic response, which is energetically costly and can disrupt systemic homeostasis. However, Wang found that TCS exposure reduced mRNA expression of Er and Ar in the hepatopancreas. Specifically, the gene expression of Er and Ar was significantly downregulated in the 0.04, 0.08, and 0.16 mg/L TCS treatment groups, with the most pronounced reduction in the 0.04 mg/L treatment [30]. This discrepancy highlights that the endocrine-disrupting effects of TCS are not uniform and are likely highly dependent on specific experimental conditions. The opposing results could be attributed to differences in the target organ (liver vs. hepatopancreas), the exposure concentration, or the duration of exposure, all of which can influence the transcriptional and feedback responses of hormone receptors. Moreover, TCS exposure led to a significant increase in Vtg mRNA and protein levels in the liver of experimental carp, indicating that TCS may have estrogen-like effects and can induce Vtg expression [58]. The TCS-induced increase in Vtg levels was correlated with elevated serum E2 levels, suggesting that TCS may indirectly induce Vtg production by increasing endogenous E2 levels [30]. Quantitative real-time PCR analysis of zebrafish tissue samples exposed to TCS revealed changes in the expression levels of HPG axis-related genes [16]. Specifically, alterations were observed in hypothalamic releasing hormones (*gnrh2*, *gnrh3*), the pituitary gonadotropin subunit (*fshβ*), and key steroid and gonadotropin receptors (*erα*, *erβ*, *lhr*, *fshr*), demonstrating a multi-level disruption of the reproductive endocrine system. Transcriptome sequencing analysis indicated that TCS exposure affects sex hormone biosynthesis pathways, including steroid biosynthesis and steroid hormone biosynthesis pathways [16]. This broad dysregulation at the genomic level provides a mechanistic basis for the observed physiological effects of TCS, namely disrupted gonadal maturation and ultimately, reduced fertility.

Beyond its well-characterized effects on the reproductive (HPG) axis, TCS also poses a significant threat to thyroid homeostasis. Due to its structural similarity to thyroid hormones, TCS (3–300 μg/L) may disrupt the HPT axis function, affecting the synthesis and secretion of T3 and T4, thereby interfering with fish morphology, physiology, and metamorphic development [66,102,103,104]. TCS exposure led to an increase in follicular area in zebrafish thyroid tissue, accompanied by hyperplasia, nuclear hypertrophy, and angiogenesis. Morphological changes in thyroid follicular epithelial cells, including increased nuclear area and cell height, as well as thickening of the follicular cell layer, are classic histological indicators of impaired thyroid function. These alterations represent a compensatory hyperplastic response by the gland, which is attempting to overcome a blockade in hormone synthesis (e.g., inhibition of thyroperoxidase) and thus directly suggest that TCS acts as a thyroid-inhibiting agent [66,102,105]. It was found that TCS exposure (300 μg/L, embryos from 30 min to for up to 7 or 14 days post fertilization) reduced thyroid hormone levels in zebrafish, including total triiodothyronine, total thyroxine, free triiodothyronine, and free thyroxine [66]. Simultaneously, TCS exposure downregulated the expression of HPT axis-related genes, further disrupting thyroid hormone function. This includes genes essential for pituitary stimulation (*tshβ*), iodide uptake (*nis*), hormone synthesis (*tpo*, *tg*), and cellular signaling (*thrα*/*β*), representing a multi-level attack on the HPT axis that severely affects metamorphosis and development [103,104,105]. TCS exposure also inhibited the expression of the insulin-like growth factor-1 (IGF-1) gene, a key regulator of somatic growth, which may further impair fish growth and development [20].

Serum biochemical and metabolomic analyses revealed that TCS exposure significantly disrupts metabolic homeostasis and tissue function in fish embryos. Specifically, increases in serum glucose, aspartate aminotransferase and alanine aminotransferase, along with decreases in total protein and albumin, indicate metabolic disorders and tissue damage [38]. Meanwhile, decreased oxygen partial pressure and increased carbon dioxide partial pressure reflect impaired respiratory function and acid-base balance disturbances [17]. At the metabolite level, significant increases in urea, citric acid, D-glucose, D-galactose, stearic acid, L-proline, phenylalanine, and L-glutamate indicate disruptions in nitrogen metabolism, energy metabolism, and protein synthesis [106,107]. Elevated urea levels suggest abnormal nitrogen metabolism or impaired kidney function, while increased citric acid reflects disturbances in the TCA cycle, potentially affecting energy supply. The upregulation of key metabolic enzymes such as alanine aminotransferase and glutamate dehydrogenase in embryos indicates enhanced amino acid metabolism to compensate for energy stress induced by TCS. Increased monosaccharide levels suggest elevated energy demand or disrupted metabolic pathways, while increased amino acids may be associated with adaptive regulation of protein synthesis, neurotransmission, and antioxidant defense. Additionally, upregulation of glucose-6-phosphate dehydrogenase suggests enhanced pentose phosphate pathway activity to produce more NADPH in response to oxidative stress, while downregulation of phosphoglucomutase indicates inhibition of gluconeogenesis and glycolysis. These changes demonstrate that TCS exposure disrupts metabolic pathways at multiple levels, leading to comprehensive stress on energy supply, nitrogen metabolism, and antioxidant defense in embryos [106,107].

## 5. Reproductive Toxicity

In the reproductive system, a growing body of evidence demonstrates that TCS exposure inflicts pronounced damage on the reproductive organs of both sexes. In females, TCS induces ovarian pathologies such as ooplasmic atrophy, accumulation of proteinaceous fluid in the interstitial stroma, altered oocyte morphology, and structural abnormalities of the corona radiata, collectively impairing oocyte maturation and ovulation [89]. In males, TCS exposure not only elevates the gonadosomatic index in zebrafish [14] but also elicits histological lesions—including seminiferous-tubule fusion, hypertrophy of spermatogonia and Leydig cells, intratubular edema, and karyolysis of spermatogonia—that ultimately compromise spermatogenesis and sperm quality [14,89].

Beyond direct gonadal injury, TCS markedly perturbs sexual differentiation and reproductive function. TCS up-regulates male-promoting genes (e.g., *sox9a*, *dmrt1a*, and *amh*) while down-regulating female-promoting genes (e.g., *wnt4a*, *cyp19a1b*, *cyp19a1a*, and *vtg2*), shifting sex ratios even at environmentally relevant concentrations (2 μg/L) [21]. Male mosquitofish exposed to 350 nM TCS exhibit a pronounced upregulation of hepatic Vtg mRNA, indicating estrogen-mimetic activity that drives feminization and skews population sex ratios [101]. This apparent contradiction, where TCS can induce both masculinizing and feminizing effects, is a recognized feature of endocrine-disrupting chemicals. The divergent outcomes likely result from differences in species sensitivity, exposure concentration, and the specific biological pathways affected. This duality highlights that TCS is a multifaceted endocrine disruptor, with its net effect being highly context-dependent. Concomitant histological deterioration of ovaries and testes reduces gametogenesis and reproductive output. A decline in the gonadosomatic index following TCS treatment reflects gonadal underdevelopment and impaired fecundity [14]. Sperm counts in TCS-treated males are significantly reduced [14,101], whereas TCS-exposed females show markedly lower daily fecundity (eggs per female per day), underscoring compromised oviposition [73]. Importantly, TCS effects are not confined to exposed individuals; epigenetic or cytoplasmic factors transmitted via oocytes or sperm can precipitate transgenerational reproductive dysfunction in offspring [23].

## 6. Neurotoxicity

In the nervous system, TCS exposure has been shown to markedly disrupt neurodevelopment and function in larval zebrafish. Morphological analyses reveal that TCS treatment reduces neuron number, induces vascular malformations, impairs hair-cell development, and perturbs neuromast formation along the lateral line, accompanied by an increased V-shaped body angle and discontinuous neuromuscular junctions, ultimately leading to aberrant motor and sensory behaviors [61,93,108]. Behavioral assays further demonstrate that TCS exposure significantly alters spontaneous locomotion and the response patterns to light–dark and acoustic stimuli [109].

Mechanistic studies at the molecular level indicate that TCS dysregulates the expression of key neurodevelopmental genes—including *olig2*, *gfap*, *mbp*, *bdnf*, *ngn1*, *syn2a*, and *cd36*—that govern neuronal maturation, myelination, and synaptic function [109]. For instance, down-regulation of myelin basic protein (*mbp*) can slow nerve impulse conduction, whereas aberrant synapsin IIa (*syn2a*) expression compromises synapse formation and neurotransmitter release [60]. Moreover, TCS interferes with cholinergic and dopaminergic signaling by inhibiting acetylcholinesterase (AChE) and dopamine activity, promoting neuronal apoptosis and diencephalic injury and thereby disrupting neurotransmission and behaviors [69,79,92]. Evidence suggests that TCS can directly bind to the catalytic site of AChE, exerting competitive inhibition [61]. At the post-transcriptional level, TCS modulates microRNA expression to further impair neurodevelopment. Exposure markedly up-regulates miR-137, thereby disturbing neural stem-cell proliferation, differentiation, and synaptic maturation [65], while altered miR-219 expression impedes oligodendrocyte precursor cell differentiation into mature oligodendrocytes, resulting in defective myelination [110].

## 7. Hepatotoxicity

In the liver, TCS exposure likewise elicits pronounced toxic effects. Chronic exposure significantly elevates liver weight and the hepatosomatic index in adult zebrafish, implying hepatic injury or steatosis [6,58]. Histological examination reveals cytoplasmic vacuolation, hepatocellular dissociation, and inflammatory-cell infiltration in TCS-treated livers, indicating disruption of hepatic architecture and function [15]. Cytoplasmic vacuolation is thought to reflect compromised membrane integrity and impaired energy metabolism [51].

At the metabolic level, TCS exposure leads to massive hepatic lipid accumulation and is accompanied by elevated serum triglycerides, total cholesterol, and low-density lipoprotein cholesterol, together with decreased high-density lipoprotein cholesterol, denoting disorders of lipid metabolism [15]. Mechanistic studies show that TCS binds avidly to PPARγ and markedly up-regulates PPARγ and its downstream targets (e.g., *scd* and *fads2*) while down-regulating *lpl*, thereby disrupting lipid metabolism via PPARγ pathway activation [6,15]. Additionally, TCS exposure reduces miR-101a expression, whereas miR-101a overexpression mitigates hepatic injury and metabolic imbalance, suggesting a protective role for miR-101a [6].

Inflammatory responses also contribute to TCS-induced hepatotoxicity. Exposure increases the pro-inflammatory cytokine TNF-α and promotes formation of Mallory–Denk bodies and intracellular hyaline bodies in hepatocytes; these protein aggregates are classic histopathological markers of hepatic injury and inflammation [51]. Moreover, TCS causes excessive hepatic accumulation of vitellogenin, leading to hepatocyte hypertrophy and further compromising liver function [15]. At the epigenetic level, TCS exposure markedly decreases global m6A-RNA methylation in zebrafish liver. This reduction is associated with enhanced demethylase FTO activity, suppressed expression of the methyltransferase METTL3, and dysregulated expression of the m6A reader protein YTHDF1. Collectively, this disruption of the m6A machinery leads to widespread instability and dysregulation of messenger RNAs involved in critical liver functions, such as metabolism and detoxification. This indicates that TCS exacerbates liver injury by perturbing m6A-mediated post-transcriptional regulation [15].

## 8. Summary and Perspectives

TCS, a widely used antimicrobial agent, persists in aquatic environments and bioaccumulates in organisms, raising significant concerns regarding its ecological and human health risks. Emerging research indicates that beyond acute toxicity, TCS induces a range of complex biological effects even under chronic, low-dose exposure conditions. It disrupts mitochondrial function, resulting in excessive ROS formation, oxidative stress, and apoptosis. Concurrently, it interferes with multiple signaling pathways, leading to energy-metabolic imbalances and DNA damage. At the organismal level, TCS exposure has severe multigenerational impacts on fish. It markedly impairs embryonic development, reducing hatchability and larval survival while inducing malformations and growth restrictions. Furthermore, TCS alters sex ratios and diminishes reproductive capacity in adulthood. Critically, these effects are dose- and time-dependent; even environmentally relevant concentrations can elicit significant developmental and reproductive toxicity, underscoring that the risks of TCS extend well beyond those identified by traditional acute toxicity evaluations.

The adverse impacts of TCS are cross-hierarchical and multi-systemic. In the liver, it disrupts hepatic structure and function, precipitating lipid metabolic disorders and inflammatory responses. Within the immune system, TCS disturbs the balance of pro- and anti-inflammatory cytokines, thereby weakening host defenses. In the nervous system, it induces neuronal apoptosis, impairs myelination, and alters behavioral patterns. Finally, for reproductive health, TCS causes histopathological damage in gonadal tissues, modulates genes involved in sex determination, and ultimately reduces fecundity and gamete quality. Notably, growing evidence suggests that TCS acts not only through established pathways of endocrine disruption and oxidative stress, but also via epigenetic mechanisms—including DNA methylation, miRNA regulation, and m6A RNA modification—which can reprogram gene expression. Such epigenetic alterations may lead to functional impairments in exposed individuals and can be transmitted to subsequent generations via gametes, resulting in transgenerational phenotypic effects that significantly amplify ecological risks. Critically, this potential for heritable, epigenetic damage challenges current risk assessment frameworks, which largely focus on direct, acute toxicity, and underscores the urgent need for regulatory policies to account for these long-term, multigenerational hazards.

From an ecological standpoint, the persistence and bioaccumulation of TCS in water, sediment, and biota facilitate its trophic transfer through food webs, threatening the sustainability of fish populations. This biomagnification poses a direct human health risk, as TCS enters the human food chain primarily through the consumption of contaminated fish and seafood, potentially exposing higher trophic levels to concerning concentrations. For human health, TCS exposure has been associated with thyroid dysfunction, metabolic disorders, and reduced fertility. Its multi-route exposure via personal care products, drinking water, and food constitutes a considerable public health concern. These findings represent a considerable public health concern, necessitating stricter regulatory measures such as limiting the use of TCS in consumer products and enhancing environmental monitoring to protect ecosystem and human health.

Despite recent advances, critical knowledge gaps remain. The current understanding of TCS toxicity, while substantial, is constrained by several critical gaps that limit a complete ecological risk assessment. Foremost among these is the scarcity of long-term, multigenerational studies that reflect realistic, low-dose exposure scenarios, which is crucial for uncovering chronic health impacts and true population-level threats. Furthermore, the mechanistic basis of TCS action remains partially elucidated, particularly concerning its epigenetic influences and the potential for these changes to cause stable, transgenerational effects. Finally, the environmental reality of TCS co-occurring with a mixture of other pollutants is poorly represented in toxicological studies, leaving a significant blind spot regarding potential synergistic interactions that could amplify its toxicity. Addressing these interconnected gaps—through integrated multi-omics approaches and multi-stressor experimental designs—is essential for developing accurate risk models and effective regulatory policies.

Moving forward, research on TCS should pursue several key directions: (1) Elucidate core mechanisms: mechanistic studies must further dissect mitochondrial dysfunction, pathway interactions, and the integrated impacts on the immune and nervous systems to identify critical molecular targets. (2) Decipher epigenetic regulation: epigenetic analyses provide a promising framework for understanding chronic and transgenerational risks and warrant integrated transcriptomic, methylomic, and non-coding RNA investigations. (3) Enhance ecological relevance: ecological and environmental studies should prioritize long-term, low-dose, and multi-pollutant exposure regimes, combining laboratory data with field monitoring to improve risk assessment accuracy. In summary, TCS exemplifies how a potential endocrine disruptor can elicit effects spanning from acute toxicity to chronic, systemic, and transgenerational impairments, highlighting the profound hazards pollutants pose across multiple levels of biological organization. Effectively addressing this complex threat demands a deeply interdisciplinary approach, integrating the expertise of molecular toxicologists, ecologists, geneticists, and environmental modelers. This collaborative framework is essential to leverage advances in detection technologies, omics platforms, and systems biology, ultimately elucidating the complete mechanistic and risk profile of TCS and providing the robust scientific foundation needed to protect environmental and human health.

## Figures and Tables

**Table 1:** Reported concentrations of triclosan in water and sediments across different regions.

Location	Matrix	Concentration	Reference
Six lakes and Nag River, Nagpur, India	Water	74.3 μg/L	[16]
Gomati River, South India	Water	1.1–9.65 μg/L (Maximum: 9.56 μg/L)	[17,18]
Freshwater lake, Yangtze River Basin, Central China	Water	0.47 μg/L	[17]
Baiyangdian Lake, China	Water	26.1 ng/L	[19]
Laizhou Bay, China	Water	58.3 ng/L	[20]
Bouregreg River, Rabat, Morocco	Water	48–301 ng/L	[20]
Yangtze River, China	Water	1.0–20.6 ng/L	[20]
Yamuna River, India	Water	269.8 ± 1.0 ng/L	[20]
Lake Victoria, Uganda	Water	89–1400 ng/L	[20]
Tamiraparani River, India	Water	Up to 5.2 μg/L	[21]
Huangpu River, China	Water	1.48–89.76 ng/L	[22]
Shanghai, China	Water	533–774 ng/L	[23]
Benedict River and lakes, India	Water	297–1761 ng/L	[24]
Tamiraparani River, India	Water	0.944 μg/L	[25]
Kaveri and Vellar Rivers, India	Water	3.8–5.16 μg/L	[25]
Xiaoqing River, China	Water	Up to 245 ng/L	[26]
Torsa River, India	Water	0.055–0.184 μg/L	[27]
Buffalo River, Eastern Cape, South Africa	Water	0–1264.2 ng/L	[28]
Estuarine system, Bangkok, Thailand	Water	Up to 185 ng/L	[29]
Freshwater, Switzerland	Water	18–98 ng/L	[2]
Yellow River, China	Water	Up to 64.7 ng/L	[30]
Atibaia River, São Paulo, Brazil	Water	Up to 0.34 mg/L	[31]
Paraíba do Sul River, São Paulo, Brazil	Water	Up to 0.78 mg/L	[31]
Pearl River Delta, South China	Water	1 μg/L	[32]
Campredo Lake, Spain	Water	0.285 μg/L	[32]
Hunting Creek, USA	Water	15.5 ± 3.71 ng/L	[33]
Ikpa River Basin, Nigeria	Water	55.1–297.7 ng/L	[34]
Jiulong River and estuary, China	Water	64 ng/L	[35]
Ton Canal, Japan	Water	134 ng/L	[35]
Ems estuary, Germany	Surface water	0.012–0.11 ng/L	[35]
Weser estuary, Germany	Surface water	0.018–0.620 ng/L	[35]
Elbe estuary, Germany	Surface water	1.20–6.87 ng/L	[35]
Vellar River, India	Surface water	Up to 516 μg/L	[36]
Tamiraparani River, Tamil Nadu, India	Surface water	Mean: 944 ng/L; Maximum: 5160 ng/L	[14]
United States	Surface water	250–850 ng/L	[18]
England	Surface water	58 μg/L	[20]
Guangzhou, China	Tap water	14.5 ng/L	[26]
Hunting Creek, USA	Sediment	72.5 ± 9.41 ng/g (d.w.)	[33]
Estuarine system, Bangkok, Thailand	Sediment	242 ng/g	[29]
Valiyar estuary, India	Sediment	132–3073 μg/kg	[25]
Baiyangdian Lake, China	Sediment	32.5 ng/g	[19]
Pearl River, China	Sediment	1329 μg/kg	[16]
Gomati River, South India	Sediment	5.11–50.36 μg/kg	[37]
Valiyar estuary, Tamil Nadu, India	Sediment	132–3073 ng/kg	[14]
Freshwater, Thailand	Sediment	Up to 0.726 mg/kg	[38]
Municipal WWTP, Savannah, USA	Effluent	86.161 μg/L	[23]

**Table 2:** Reported LC_50_ values for various fish species following exposure to triclosan.

Test Organism	Exposure Duration	LC_50_	Reference
*Aphaniops hormuzensis*	96 h	0.924 mg/L	[55]
*Carassius auratus*	96 h	1111.9 µg/L, 1.839 mg/L	[56,57]
*Catla catla*	96 h	0.73 mg/L	[58]
*Catla catla*	96 h	0.36 mg/L	[3]
*Cirrhinus mrigala*	96 h	0.131 mg/L	[46]
*Clarias gariepinus*	96 h	16.04 mg/L	[52]
*Ctenopharyngodon idella*	96 h	0.116 mg/L	[46]
*Cyprinus carpio*	96 h	0.80 mg/L	[30]
*Cyprinus carpio*	96 h	0.80 mg/L	[56]
*Cyprinus carpio*	96 h	0.315 mg/L	[46]
Zebrafish embryos (*Danio rerio*)	120 h	217 μg/L	[59]
Zebrafish embryos (*Danio reri*o)	96 h	267.8 μg/L, 0.608 ± 0.064 mg/L, 420 μg/L	[20,60,61,62]
Zebrafish embryos (*Danio rerio*)	48 h	1.50 ± 0.48 mg/L	[63]
Juvenile zebrafish (*Danio rerio*)	96 h	510 μg/L, 0.42 mg/L	[64,65,66,67,68]
*Danio rerio*	96 h	340 mg/L, 398.9 μg/L	[60,62,66,69]
*Danio rerio*	32 days	74.6 μg/L	[20]
*Gambusia affinis*	96 h	1.399 mg/L	[51]
*Labeo rohita*	96 h	126 μg/L	[25]
*Labeo rohita*	96 h	96 μg/L, 0.39 mg/L	[2,20,45]
*Misgurnus anguillicaudatus*	96 h	0.045 mg/L	[57]
*Oreochromis mossambicus*	96 h	715 μg/L, 740 μg/L	[20,70]
*Oryzias latipes*	96 h	1.7 mg/L, 210 mg/L	[71,72]
*Oryzias latipes*	21 days	330.6 μg/L	[73]
*Oryzias latipes*	96 h	169.78 μg/L, 399 μg/L	[73,74]
*Oryzias latipes*	96 h	117.9, 600, 602 µg/L	[73,74]
*Oryzias latipes*	48 h	0.352 mg/L	[35]
*Oryzias latipes*	96 h	1700 μg/L	[75]
*Oryzias melastigma*	-	300 μg/L	[20]
*Oryzias sinensis*	96 h	0.63 mg/L	[76]
*Pangasianodon hypophthalmus*	96 h	848.33 μg/L (25 °C) 1181.94 μg/L (30 °C) 1356.96 μg/L (35 °C)	[77]
*Pangasianodon hypophthalmus*	96 h	910 mg/L (pH 6.5) 1110 mg/L (pH 7.5) 1380 mg/L (pH 8.5)	[78]
*Pangasianodon hypophthalmus*	96 h	1458 μg/L	[79]
*Pangasianodon hypophthalmus*	96 h	1177 μg/L	[80]
*Pimephales promelas*	96 h	260 μg/L	[75,81]
*Poecilia vivipara*	96 h	0.6 mg/L	[82]
*Pseudorasbora parva*	96 h	0.071 mg/L	[57]
*Tanichthys albonubes*	96 h	0.889 mg/L	[57]
*Xiphophorus helleri*	96 h	1.47 mg/L	[83]
*Lepomis macrochirus*	96 h	370 µg/L	[81]

**Table 3:** Reported NOEC values for various fish species following exposure to triclosan.

Test Organism	Exposure Duration	NOEC	References
Zebrafish (*Danio rerio*) embryos	96 h	200 μg/L	[84]
Zebrafish (*Danio rerio*) embryos	144 h	160 μg/L	[19]
Zebrafish (*Danio rerio*) embryos	144 h	160 μg/L	[36]
Zebrafish (*Danio rerio*)	9 days	26 μg/L	[20]
Zebrafish (*Danio rerio*)	32 days	48.4 μg/L	[20]
Rainbow trout (*Oncorhynchus mykiss*)	35 days	71.3 μg/L	[20]
Medaka (*Oryzias latipes*)	182 days	11 μg/L	[54]

## Data Availability

No new data were created or analyzed in this study.
